# Differential Metabotypes in Synovial Fibroblasts and Synovial Fluid in Hip Osteoarthritis Patients Support Inflammatory Responses

**DOI:** 10.3390/ijms23063266

**Published:** 2022-03-17

**Authors:** Hussein Farah, Susanne N. Wijesinghe, Thomas Nicholson, Fawzeyah Alnajjar, Michelangelo Certo, Abdullah Alghamdi, Edward T. Davis, Stephen P. Young, Claudio Mauro, Simon W. Jones

**Affiliations:** 1 Institute of Inflammation and Ageing, MRC Versus Arthritis Centre for Musculoskeletal Ageing Research, College of Medical and Dental Sciences, University of Birmingham, Birmingham B15 2TT, UK; hxf820@student.bham.ac.uk (H.F.); s.n.wijesinghe@bham.ac.uk (S.N.W.); t.a.nicholson@bham.ac.uk (T.N.); fxa790@student.bham.ac.uk (F.A.); m.certo@bham.ac.uk (M.C.); axa1316@student.bham.ac.uk (A.A.); s.p.young@bham.ac.uk (S.P.Y.); c.mauro@bham.ac.uk (C.M.); 2 The Royal Orthopaedic Hospital, NHS Foundation Trust, Bristol Road South, Northfield, Birmingham B31 2AP, UK; edward.davis@nhs.net

**Keywords:** osteoarthritis, synovial fibroblast, glutamine, IL6, metabolism, inflammation

## Abstract

Changes in cellular metabolism have been implicated in mediating the activated fibroblast phenotype in a number of chronic inflammatory disorders, including pulmonary fibrosis, renal disease and rheumatoid arthritis. The aim of this study was therefore to characterise the metabolic profile of synovial joint fluid and synovial fibroblasts under both basal and inflammatory conditions in a cohort of obese and normal-weight hip OA patients. Furthermore, we sought to ascertain whether modulation of a metabolic pathway in OA synovial fibroblasts could alter their inflammatory activity. Synovium and synovial fluid was obtained from hip OA patients, who were either of normal-weight or obese and were undergoing elective joint replacement surgery. The synovial fluid metabolome was determined by 1H NMR spectroscopy. The metabolic profile of isolated synovial fibroblasts in vitro was characterised by lactate secretion, oxygen consumption rate (OCR) and extracellular acidification rate (ECAR) using the Seahorse XF Analyser. The effects of a small molecule pharmacological inhibitor and siRNA targeted at glutaminase-1 (GLS1) were assessed to probe the role of glutamine metabolism in OA synovial fibroblast function. Obese OA patient synovial fluid (n = 5) exhibited a different metabotype, compared to normal-weight patient fluid (n = 6), with significantly increased levels of 1, 3-dimethylurate, N-Nitrosodimethylamine, succinate, tyrosine, pyruvate, glucose, glycine and lactate, and enrichment of the glutamine–glutamate metabolic pathway, which correlated with increasing adiposity. In vitro, isolated obese OA fibroblasts exhibited greater basal lactate secretion and aerobic glycolysis, and increased mitochondrial respiration when stimulated with pro-inflammatory cytokine TNFα, compared to fibroblasts from normal-weight patients. Inhibition of GLS1 attenuated the TNFα-induced expression and secretion of IL-6 in OA synovial fibroblasts. These findings suggest that altered cellular metabolism underpins the inflammatory phenotype of OA fibroblasts, and that targeted inhibition of glutamine–glutamate metabolism may provide a route to reducing the pathological effects of joint inflammation in OA patients who are obese.

## 1. Introduction

Osteoarthritis (OA) is usually seen in older individuals and thus has long been regarded as a “wear and tear” disease in contrast to the immune driven, systemic inflammatory joint disease rheumatoid arthritis (RA). However, increasingly, OA is now being regarded as a localised inflammatory joint disease [1,2,3], in which the inflammation contributes significantly to disease progression by driving degradation of articular cartilage [4,5] and also promotes symptomatic pain [6,7,8]. Synovitis, typified by synovial lining hyperplasia, inflammatory cell infiltration [9] and stromal vascularisation [10,11], is present in OA patients early in the disease course [9] prior to radiographic signs of cartilage damage, suggesting synovitis is amongst the early pathophysiological changes in OA.

Despite this evidence base, clinical trials on anti-inflammatory drugs such as TNFα monoclonal antibodies or IL-1β antagonists have produced disappointing results [12]. One reason for this is likely the lack of stratification in these clinical trials, with patients not selected for exhibiting overt synovitis. Indeed, we have previously reported that OA patients who are obese exhibit a more inflammatory synovial fluid profile than OA patients who are of normal-weight [13,14], and therefore could be more amenable to such anti-inflammatory therapeutic approaches. However, it is also true that trials of anti-inflammatory drugs in OA patients have involved the testing of repurposed drugs, which have proved successful in patients with other arthridies, such as RA, rather than drugs that have been specifically designed on the basis of modifying OA disease pathology [12]. To this end, there is a clear need to better understand the underlying pathways that mediate OA synovitis in order to identify disease specific candidate targets and pathways for therapeutic modulation.

Importantly, we and others have reported that the inflammatory phenotype of the OA synovial fibroblast is a central contributor to the inflammatory environment of the synovial joint fluid [13,14], and that distinct cellular subsets are associated with joint pain [6]. Therefore, identifying pathways and targets that modulate the inflammatory phenotype of the synovial fibroblast may represent a rewarding therapeutic strategy to develop an efficacious OA disease-modifying anti-inflammatory drug. Notably, recent evidence across multiple chronic inflammatory conditions suggests a relationship between the inflammatory activated phenotype of tissue resident stromal fibroblasts and their metabolic phenotype [15]. For example, in renal models, interstitial fibroblasts exhibit decreased mitochondrial respiration and increased expression of glycolytic enzymes [16]. Similarly, in pulmonary fibrosis, activated myofibroblasts exhibit increased expression of the glycolytic enzyme phosphofructo-2-kinase/fructose-2,6-biphosphatase 3 enzyme (PFKFB3), and use of a PFKFB3 inhibited attenuated the differentiation of lung fibroblasts into activated fibroblasts [17]. Notably, there is now evidence that similar metabolic changes could underpin inflammatory joint disease. In RA, metabolic profiling of synovial fibroblasts has implicated alterations in glycolytic metabolism [18,19], glutamine metabolism [20], and the pentose phosphate pathway [21] to synovial proliferation and inflammatory phenotype.

Importantly, in the OA joint, we have reported that obesity imprints an inflammatory activated synovial fibroblast phenotype, with fibroblasts from obese OA patients being more proliferative and secreting greater amounts of IL-6 [13,14]. Importantly, obesity is a major risk factor for the development of OA in both weight-bearing joints such as the knee [22,23] and hip [24], as well as non-weight bearing joints such as the hand [25], promoting cartilage degeneration [26] and sclerotic subchondral bone pathology [27]. Therefore, understanding whether metabolic differences underpin this obese inflammatory activated OA synovial fibroblasts could provide a new rationale for targeting synovial joint inflammation in OA. The aim of this study was therefore firstly to examine whether synovial fluid and synovial fibroblasts from obese inflammatory OA patients exhibit differential metabolic pathotypes, compared to lean normal-weight OA patients—secondly, to determine whether pharmacological modulation of metabolism in synovial fibroblasts can modify their inflammatory phenotype.

## 2. Results

### 2.1. NMR Spectroscopy Reveals Differential Synovial Fluid Metabotypes in Obese and Normal Weight Hip OA Patients

**Figure 1:**
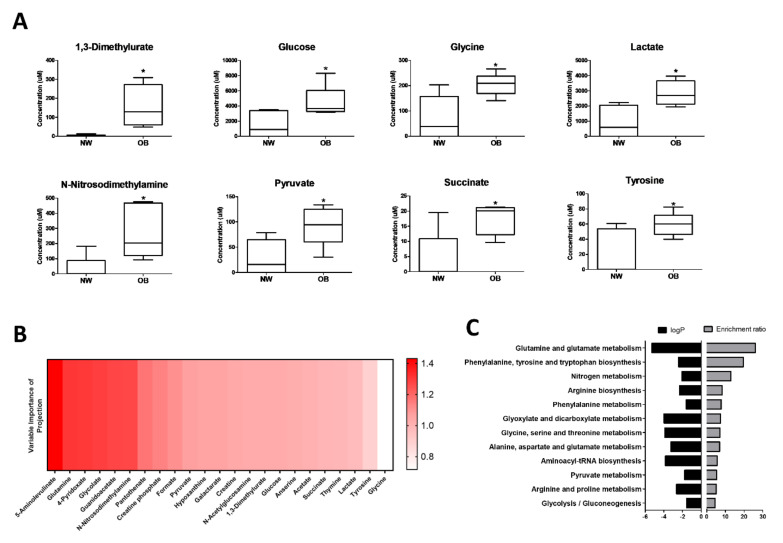
NMR spectroscopy reveals different metabotypes between obese and normal-weight OA patient synovial fluid (**A**) Concentration (μM) of selected metabolites that was found to be significantly different in synovial fluid from obese (OB; n = 5) and normal-weight (NW; n = 6) OA patient synovial fluids. * = *p* < 0.05; (**B**) top metabolites ranked on Variable Importance in Projection (VIP) score between obese (OB; n = 5) and normal-weight (NW; n = 6) OA synovial fluid; (**C**) enrichment analysis using MetaboAnalyst, of metabolites with VIP scores ≥1 between obese and normal-weight synovial fluids, showing enrichment ratio and associated *p*-values for the most relevant metabolic pathways.

Synovial fluids were collected from n = 5 obese and n = 6 normal-weight OA patients (patient characteristics in Table 1) for analysis by NMR spectroscopy. In total, the presence of 133 metabolites were detected, of which eight were found to be significantly increased (*p* < 0.05, FC > 1.5) in obese synovial fluids, compared to normal-weight synovial fluids (Figure 1A, Supplementary Table S1). These were 1,3-Dimethylurate, Glucose, Glycine, Lactate, N-Nitrosodimethylamine, Pyruvate, Succinate and Tyrosine. Subsequent linear regression analysis with parameters of body composition found that synovial fluid concentrations of Glycine (R^2^ = 0.58, *p* < 0.01), Lactate (R^2^ = 0.41, *p* < 0.05), Succinate (R^2^ = 0.50, *p* < 0.05), and Tyrosine (R^2^ = 0.39, *p* < 0.05) were positively correlated with increasing BMI. The relationships between these metabolites and increasing adiposity were strengthened when comparing to waist:hip ratio (WHR) as a measure of central adiposity, with concentrations of Glycine (R^2^ = 0.66, *p* < 0.01), Lactate (R^2^ = 0.90, *p* < 0.001), Succinate (R^2^ = 0.52, *p* < 0.05), Tyrosine (R^2^ = 0.52, *p* < 0.05) and also N-Nitrosodimethylamine (R^2^ = 0.51, *p* < 0.05) were positively correlated with increasing WHR (Supplementary Table S2).

Subsequent multivariate analyses of metabolites were conducted to generate Variable Importance in Projection (VIP) scores to estimate the importance of each metabolite in modelling the difference between synovial fluid metabolomic profiles based on BMI. The metabolite with the highest VIP score in the comparison between obese and normal-weight synovial fluids was 5-aminolevulinate, followed by glutamine (Figure 1B). Notably, KEGG enrichment analysis of metabolites with VIP ≥ 1 identified glutamine–glutamate metabolism as the metabolic pathway with the highest enrichment ratio and the most significantly altered pathway in obese OA synovial fluid, compared to normal-weight OA synovial fluid (Figure 1C). Furthermore, the ratio of glutamine:glutamate concentration in the synovial fluid was negatively correlated (R^2^ = 0.55, *p* < 0.05) with increasing waist circumference (Supplementary Table S2).

### 2.2. Lactate Secretion Is Greater in Obese OA Synovial Fibroblasts and Is Induced during the Inflammatory Response

Previously, we and others have reported that the accumulation of lactate in the tissue microenvironment is a feature of chronic inflammatory diseases [28,29,30]. Therefore, having found that the concentration of lactate was significantly 3-fold greater in obese OA synovial fluid, compared to normal-weight synovial, we next examined whether this was reflected in the secretome of cultured human OA synovial fibroblasts since the inflammatory profile of OA fibroblasts has previously been shown to reflect the synovial fluid profile [13].

**Figure 2:**
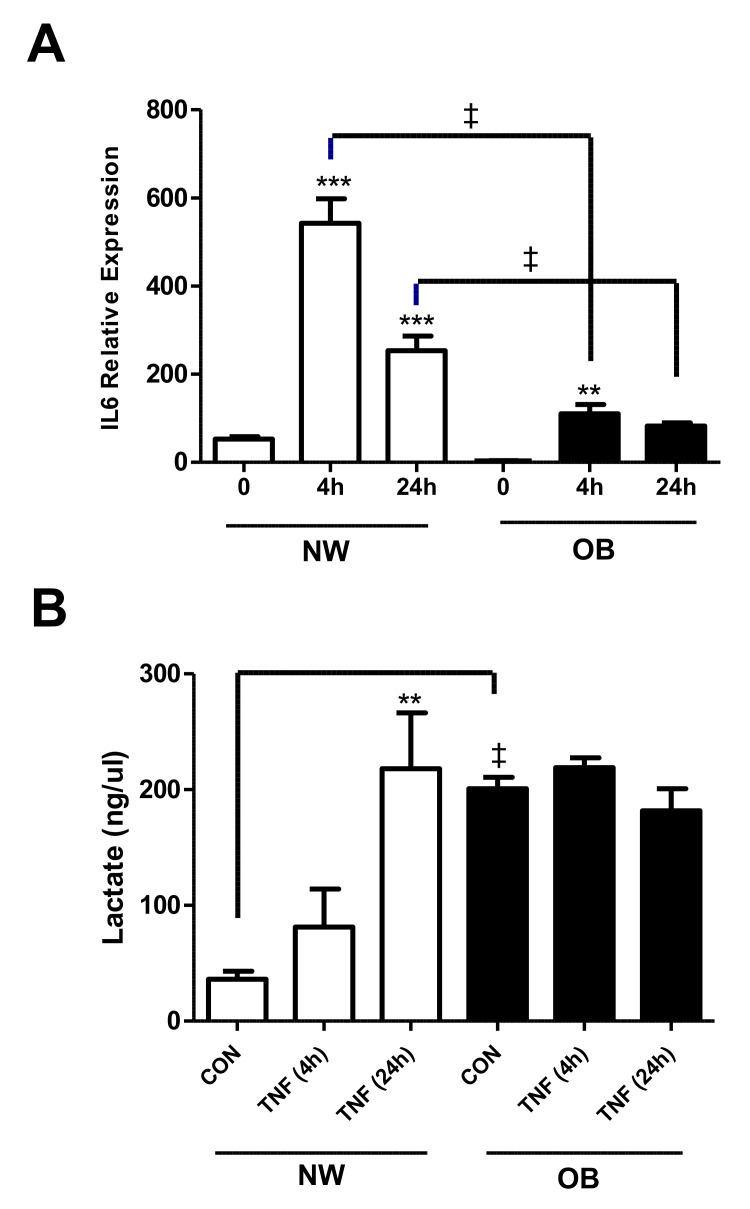
Lactate secretion is greater in obese OA synovial fibroblasts and is induced during the inflammatory response. The effect of TNFα (10 ng/mL) stimulation of synovial fibroblasts from normal-weight (NW) or obese OA patients (n = 3) on (**A**) the expression of IL-6 and (**B**) secretion of lactate after 4 h and 24 h stimulation. Data expressed as mean ± SEM. ** = *p* < 0.01, *** = *p* < 0.001, significantly different from time 0. ‡ = *p* < 0.01, significantly different between synovial fibroblasts from patients with different BMI.

Lactate production (as a measure of glycolytic flux) was measured in obese and normal-weight synovial fibroblasts under both basal and inflammatory conditions. First, to confirm that the OA synovial fibroblasts were responsive to TNFα stimulation, fibroblasts from normal weight and obese samples (n = 3) were cultured with or without TNFα for 4 and 24 h. Fibroblasts from all patient cohorts showed a significant increase in IL-6 expression at 4 h or 24 h in response to TNFα stimulation, which was most pronounced in normal-weight fibroblasts, indicating induction of a robust inflammatory response (Figure 2A). Analysis of basal (unstimulated) secretion revealed that obese fibroblasts exhibited significantly greater production of lactate, than fibroblasts from normal-weight patients (Figure 2B). Furthermore, in contrast to the normal-weight fibroblasts, stimulation of obese fibroblasts with TNFα for either 4 h or 24 h did not induce any further increase in lactate secretion (Figure 2B), suggesting that lactate production in these cells had reached maximal levels. 

### 2.3. Obese OA Synovial Fibroblasts Increase Mitochondrial Respiration When Stimulated with Pro-Inflammatory Cytokine TNFα and Show Increased Aerobic Glycolysis Compared to OA Synovial Fibroblasts from Normal Weight Patients

**Figure 3:**
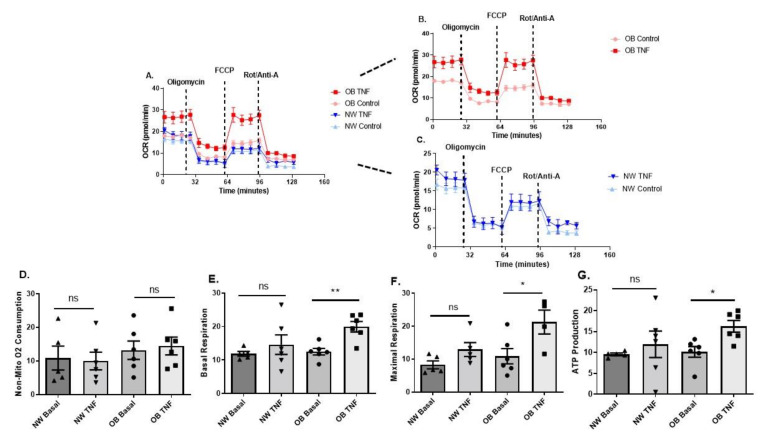
Obese and normal-weight OA synovial fibroblasts exhibit differential mitochondrial respiration. (**A**–**C**) OA synovial fibroblasts from obese patients (n = 6 patients, with 5 biological replicates per patient) stimulated with TNFα at 10 ng/mL for 24 h show increased mitochondrial respiration, compared to fibroblasts from normal weight patients. (**D**) Non-mitochondrial oxygen consumption does not increase in OA synovial fibroblasts from obese or normal weight patients with TNFα stimulation. (**E**) basal respiration; (**F**) maximal respiration; and (**G**) ATP production from mitochondrial respiration significantly increase in obese OA synovial fibroblasts when stimulated with TNFα at 10 ng/mL for 24 h. All data presented as mean ± SEM. * = *p* < 0.05, ** = *p* < 0.01.

**Figure 4:**
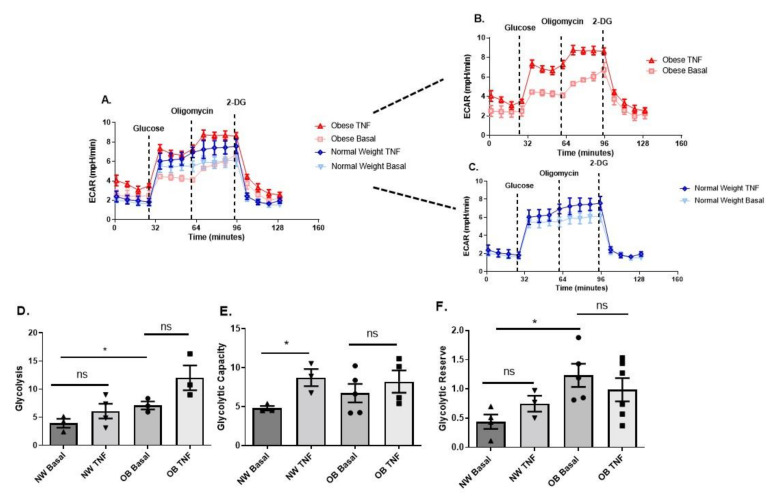
Obese and normal-weight OA synovial fibroblasts exhibit differential glycolytic metabolism. (**A**–**C**) OA synovial fibroblasts from obese patients (n = 6 patients) stimulated with TNFα at 10 ng/mL for 24 h show elevated aerobic glycolysis, compared to stimulated and unstimulated OA synovial fibroblasts from normal weight (n = 6 patients, with 5 biological replicates per patient). (**D**) Glycolysis levels are significantly increased in obese OA synovial fibroblasts when stimulated with TNFα at 10 ng/mL for 24 h. (**E**) Glycolytic capacity of normal weight OA synovial fibroblasts is elevated when challenged with TNFα at 10 ng/mL for 24 h. (**F**) Glycolytic reserve is increased in unstimulated obese OA synovial fibroblasts, compared to unstimulated normal weight OA synovial fibroblasts. All data presented as mean ± SEM. * = *p* < 0.05.

Next, we characterised the metabolic activity of synovial fibroblasts from obese and normal-weight OA patients by determining mitochondrial respiration (Figure 3A–G) and glycolysis (Figure 4A–F) in real-time using a Seahorse XFe96 Analyser. 

There was no significant difference in non-mitochondrial respiration between either normal-weight or obese fibroblasts, or between basal and TNFα stimulated fibroblasts (Figure 3D). However, in contrast to normal-weight fibroblasts, both basal and maximal mitochondrial respiration were significantly increased in obese OA synovial fibroblasts upon stimulation with TNFα for 24 h (Figure 3D,E). Furthermore, a significant increase in ATP production was observed in obese OA synovial fibroblast stimulated with TNFα, but not in similarly treated normal-weight fibroblasts (Figure 3G). 

Analysis of glycolytic metabolism revealed that obese OA synovial fibroblasts also exhibited significantly greater basal glycolysis and glycolytic reserve, compared to basal normal-weight fibroblasts (Figure 4A–D,F). However, unlike the obese fibroblasts, normal-weight fibroblasts were capable of increasing glycolytic capacity when stimulated with TNFα (Figure 4F).

### 2.4. Glutaminase-1 Inhibition Attenuates the OA Synovial Fibroblast Inflammatory Response

Having identified glutamine–glutamate metabolism as the most significantly enriched metabolic pathway between both obese and normal-weight patient synovial fluid, we next investigated whether modulation of this metabolic process altered the inflammatory phenotype of OA synovial fibroblasts. To this end, we utilised a non-competitive pharmacological inhibitor of Glutaminase-1 (GLS1), known as Bis-2-(5-phenylacetamido-1,3,4-thiadiazol-2-yl) ethyl sulfide (BPTES), thus preventing the conversion of glutamine to glutamate. OA synovial fibroblasts were cultured for 24 h either in normal growth media, or stimulated with TNFα (10 ng/mL) or stimulated with TNF (10 ng/mL) in combination with the GLS1 inhibitor BPTES (20 μM).

**Figure 5:**
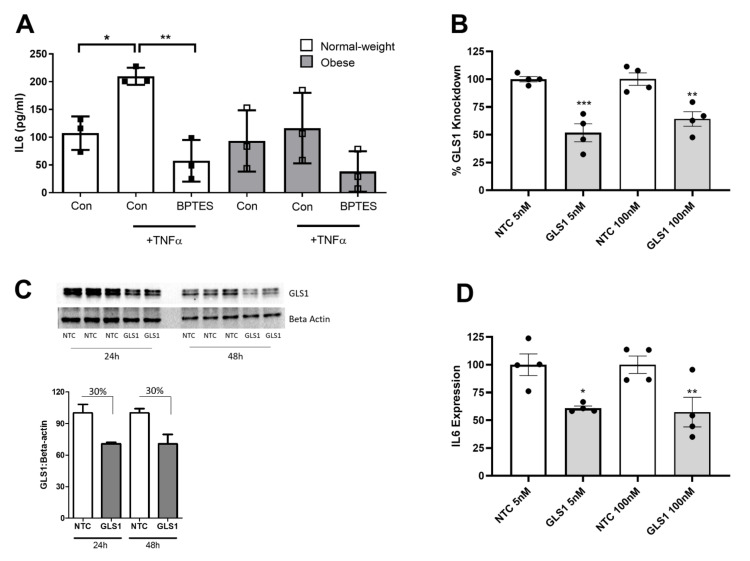
Inhibition of glutaminase-1 attenuates the IL-6 inflammatory response in OA synovial fibroblasts. (**A**) GLS1 inhibitor, BPTES (20 µM), attenuates TNFα induced IL-6 secretion in synovial fibroblasts from normal weight and obese hip OA patients (n = 3 patients). Bars represent mean IL-6 secretion (pg/mL). (**B**) % knockdown of GLS1 expression in OA synovial fibroblasts after 24 h post-transfection with a GLS1 targeting siRNA (5 nM or 100 nM), compared to a non-targeting control (NTC) siRNA (n = 4 patients). (**C**) Confirmation of siRNA-mediated GLS1 protein knockdown at 24 h and 48 h post-transfection using 100 nM siRNA. (**D**) Effect of sRNA-mediated knockdown of GLS1 on IL-6 expression in OA synovial fibroblasts at 24 h post-transfection (n = 4 patients). Data are presented as mean ± SEM. * = *p* < 0.05, ** = *p* < 0.01, *** = *p* < 0.001.

As expected, cells stimulated with TNFα exhibited a significant increase in IL-6, compared to unstimulated cells. However, cells stimulated with TNFα in the presence of the GLS1 inhibitor exhibited no increase in IL-6 release (Figure 5A). To validate the effect of the inhibitor, we next conducted a loss-of-function study using siRNA directed against GLS1. Transfection of fibroblasts with GLS siRNA for 24 h at 5 nM and at 100 nM significantly reduced GLS1 expression by 65% and 35%, respectively, compared to transfection with a non-targeting control (NTC) siRNA (Figure 5B), when normalised to an 18S housekeeping gene. Significant depletion of GLS1 expression was also observed upon normalising to a β-actin housekeeping gene (Supplementary Figure S1). Knockdown of GLS1 was also confirmed at the protein level following both 24 h and 48 h post-transfection (Figure 5C). Notably, cells depleted of GLS1 exhibited significantly reduced IL-6 expression by between 40–65%, supporting the role of GLS1 in mediating IL-6 production (Figure 5D), when normalised to 18S, and also when normalising to β-actin (Supplementary Figure S1).

## 3. Discussion

In this study, we provide evidence for metabolic differences underpinning the inflammatory profile of OA synovial fluid and the inflammatory phenotype of OA synovial fibroblasts, and that pharmacological modulation of the glutamine–glutamate pathway mediates the IL6 inflammatory response in OA synovial fibroblasts. 

Previously, the synovial fluid of hip OA patients who are obese has been reported to be more inflammatory, with increased levels of the pro-inflammatory cytokines IL6, CXCL8 and TNFα, compared to synovial fluid from OA patients of normal-weight [13]. In the present study, NMR spectroscopy revealed that differences between obese and normal-weight OA synovial fluid extend to the metabolome, with significant differences in the concentration of a number of key metabolites that regulate fuel utilisation, including succinate, pyruvate, glucose and lactate that also were positively correlated with increasing BMI and central adiposity. Notably, lactate has been reported to accumulate in tissue microenvironments in chronic inflammatory diseases, and has been suggested to be a key factor in driving immune-mediated inflammation via its uptake into CD4^+^ T-cells and the subsequent induction of their effector phenotype and retention in inflamed tissues [28,29]. Therefore, its increase presence in obese OA synovial fluid, compared to normal-weight OA synovial fluid, may indicate increased CD4^+^ T cell effector function and retention in the obese OA synovial joint. Previous NMR spectroscopy has compared OA synovial fluid to the more inflammatory synovial fluid from patients with RA, where both pyruvate and glucose were found to be significantly different [31], supporting the relationship between the presence of these metabolites and the differences in the degree of joint inflammation. 

Functional characterisation of the metabolic profile in hip OA synovial fibroblasts revealed that fibroblasts from obese patients not only exhibited increased basal secretion of lactate, compared to normal-weight fibroblasts, but also exhibited greater mitochondrial respiration, potentially supported by the observed increased the glutamine–glutamate pathway, and greater glycolytic reserve. However, upon inflammatory challenge with TNFα, obese synovial fibroblasts, unlike normal-weight fibroblasts, were not capable of secreting more lactate or being able to further increase their glycolytic reserve. 

This reduced plasticity of obese synovial fibroblasts to respond metabolically to the increased energy demands during an inflammatory challenge could play a role in mediating a pathological joint inflammation, and suggests an intimate link between the inflammatory state of cells and their metabolic status. Indeed, RA synovial fibroblasts, cancer-associated fibroblasts (CAFs) and activated myofibroblasts have all previously been shown to increase their rate of glycolysis and increase their expression of key glycolytic enzymes, such as Phosphofructokinase (PFKL), PFKFB3 and phosphoglycerate kinase 1 (PGK1) when in inflammatory micro-environments, in order to meet the energy demands of cell [15,17,18,19,32]. Therapeutically targeting dysregulated metabolic pathways may therefore provide a novel route to regulating the pathological inflammatory response in an inflammatory environment such as the osteoarthritis joint. To this end, our finding that we could reduce the IL-6 inflammatory response in OA synovial fibroblasts by targeting glutamine–glutamate metabolism using either a pharmacological inhibitor or an siRNA targeting GLS1 is notable. Previously, it has been shown that GLS1 inhibition reduces the proliferation of RA synovial fibroblasts, and in vivo reduces joint swelling in a murine model of RA [20]. Furthermore, in myofibroblasts, GLS1 inhibition reduced ECM production [33,34], a feature of synovial fibrosis in both OA [35,36,37] and RA [38] disease. Importantly, our identification of glutamine–glutamate metabolism as a clinical feature that is enriched in obese OA patient synovial fluid (a patient cohort that exhibits greater levels of inflammatory cytokines), which suggests that targeting this particular metabolic pathway will be of clinical relevance. 

A pharmacological therapeutic capable of modulating the inflammatory phenotype of the OA synovial fibroblast could modify both disease progression and reduce OA patient reported joint pain. Synovial tissue inflammation in knee OA patients is associated with increased pain severity [6,39], with sites of patient-reported pain exhibiting a differential phenotype with distinct fibroblast subsets [6]. Furthermore, synovial inflammation with the increased presence of pro-inflammatory cytokines in the synovial fluid drives cartilage degeneration via the induction of matrix metalloproteases (MMPs) and aggrecanases, which mediate type II collagen and aggregan proteoglycan degradation, respectively [4,5,40,41]. Nevertheless, such therapeutic targeting approaches represent significant challenges, given both the heterogeneity of OA disease and the ubiquitous expression of most metabolic mediators. To this end, specific targeted delivery to distinct populations of inflammatory synovial fibroblasts could be required, given the evidence of the existence of synovial fibroblast subsets that differentially drive inflammation [6,42].

Finally, our data provide further evidence of the effect of obesity on the pathophysiology of the OA joint, with differences in the synovial fluid metabolome and synovial fibroblast phenotype between obese and normal-weight OA patients. This is important since a systematic review and meta-analysis of the relationship between BMI and the risk of knee OA found that the risk of knee OA was increased by 35% with a 5 kg/m^2^ increase in BMI [43], whereas weight loss in obese knee OA patients reduced joint pain [44] and slowed cartilage degeneration over a 48-month period in overweight and obese knee OA patients [45]. Therefore, advancing our understanding of how obesity affects the OA joint could help to define strategies for both pharmacological and non-pharmacological lifestyle intervention strategies. 

In summary, this study provides further evidence for an intimate link between the inflammatory and metabolic profile of OA synovial fibroblasts and synovial fluid, and, for the first time, provides the rationale for the therapeutic targeting of glutamine–glutamate metabolism to reduce the pathological effects of joint inflammation in hip OA patients who are obese.

## 4. Materials and Methods

### 4.1. Patient Samples and Synovial Fibroblast Cell Culture

Synovial tissue biopsies and synovial fluid was collected from OA patients undergoing total hip replacement surgery at Russell’s Hall Hospital, Dudley, UK or the Royal Orthopaedic Hospital, Birmingham, UK. (NRES 14/ES/1044). Patients were classified as either normal-weight (18–25) or obese (>30) based on their BMI. For the isolation of synovial fibroblasts, synovial tissue was dissected using a scalpel into 1 mm^3^ pieces, placed into a tissue culture flask and incubated at 37 °C, 5% CO_2_ in complete fibroblast growth media (RPMI-1640 media (Sigma-Aldrich, Gillingham, UK, R8758) supplemented with 10% foetal calf serum (Sigma-Aldrich, F7524), 1% non-essential amino acids (Sigma-Aldrich, M7145), 1% sodium orthopyruvate (Sigma-Aldrich, S8636), 2 mM L-glutamine (ThermoFisher Scientific, Gloucester, UK, 25030024), 1% penicillin and streptomycin (100 U/mL penicillin and 100 µg/mL streptomycin) (Sigma-Aldrich, P4333). Fibroblasts were characterised by flow cytometry. Isolated cells were resuspended in ice cold PBS containing 1% BSA for subsequent staining at 4 °C. Antibodies used were anti-CD45 (2D1, Biolegend, London, UK), anti-CD31 (WM59, Biolegend), anti-GP38 (NC-08, Biolegend) and anti-CD235a (HI264, Biolegend). Dead cells were excluding using LIVE/DEAD™ Violet Dead Cell Stain (L34955, Invitrogen, Waltham, MA, USA). Samples were acquired using BD LSR Fortessa™ X-20 Cell Analyzer. (Supplementary Figure S2). Growth media were replaced every 3–4 days, and cells passaged upon reaching 70% confluency. All experiments were performed using synovial fibroblasts between passages 2–5.

### 4.2. siRNA Transfection

OA synovial fibroblasts were seeded overnight in 48-well plates at a concentration of 40,000 cells per well. Cells were cultured in RPMI-1640 (Sigma-Aldrich, R8758) in 1% FCS (Sigma-Aldrich, F7524), sodium orthopyruvate (1%) (Sigma-Aldrich, S8636), non-essential amino acids (1%) (Sigma-Aldrich, M7145). Cells were then transfected with either a non-targeting control (NTC) siRNA (Dharmacon, D-001206-13) or a GLS1 siRNA (Dharmacon, MQ-004548-01-0005) at concentrations of either 5 nM or 100 nM. Following 24 h, the supernatants were collected for cytokine analysis by enzyme-linked immunosorbent assay (ELISA) (IL-6 DuoSet kit, R&D Systems, DY206), and cells were lysed with TRIzol reagent (Life technologies, Paisley, UK, 15596026) for RNA extraction.

### 4.3. Quantitative RT-PCR

Total RNA was extracted using TRIzol reagent (Life technologies, Paisley, UK, 15596026) following the manufacturer’s protocol. Primers (Supplementary Table S3) for the genes 18s, IL6, actin and GLS1 were designed using Primer Design3, and qRT-PCR performed in a one-step reaction (iTaq Universal One-Step; BioRad, Watford, UK #1725151). Relative expression was determined using the ΔΔCt method, followed by normalization with 18s.

### 4.4. ELISA

Quantification of IL-6 production was determined using a commercially available ELISA (IL-6 DuoSet kit, R&D Systems, DY206) following the manufacturer’s instructions. Cell supernatants were diluted 1:200 with assay buffer before being read on a BioTek EL808 microtiter plate reader (BioTek, Swindon, UK).

### 4.5. Western Blotting

Synovial fibroblasts were lysed in RIPA buffer (Life Technologies) containing Phosphatase Inhibitor Cocktail 3 (1:100, Sigma-Aldrich, P0044) and Protease Inhibitor Cocktail (1:100, Sigma-Aldrich, P8340) on ice for 20 min. Total protein was quantified by performing a Bradford assay (Bio-Rad, #5000002) and 15 μg subjected 12% SDS-PAGE, and transferred to a methanol-activated PVDF membrane (Bio-Rad, 1620177). Membranes were immuno-probed overnight at 4 °C, with a monoclonal anti-GLS1 Primary antibody (1:1000; Abcam, ab156876) or a monoclonal anti-beta-actin antibody (1:1000, Sigma Aldrich, SAB1305546), followed by incubation for 2 h at RT with an HRP-linked anti-rabbit IgG secondary antibody (1:5000; GE Healthcare, NA931) or anti-mouse IgG secondary antibody, respectively (1:5000; GE Healthcare, NA934). Blots were developed using Amersham ECL Prime (GE Healthcare, RPN2232) and imaged using a ChemiDoc MP System (Bio-Rad). Densitometry analysis of bands was performed using an open-source, public domain software package (ImageJ v1.47).

### 4.6. Seahorse Metabolic Analysis of OA Synovial Fibroblast 

The Seahorse XFe96 Analyzer (Agilent, Santa Clara, CA, USA) was used to measure oxidative phosphorylation and glycolysis through the oxygen consumption rate (OCR) and extracellular acidification rate (ECAR), respectively, in cultured OA synovial fibroblast. Cells were washed with Seahorse XF RPMI-1640 medium (Agilent, Santa Clara, CA, USA, 103576), and the microplate was warmed for 60 min in a CO_2_-free incubator before a mitochondrial and glycolytic stress test was initiated. The seahorse assay was conducted according to standard protocol with the injection of 10 mM Glucose (Sigma-Aldrich, G7021), 2 µM Oligomycin (ATP-synthase inhibitor) (Sigma-Aldrich, O4876), 5 µM cyanide-4-(trifluoromethoxy) phenylhydrazone/FCCP (oxidative phosphorylation uncoupler) (Sigma-Aldrich, C2920), 50 mM 2-Deoxy-D-glucose/2-DG (non-metabolisable glucose analog) (Sigma-Aldrich, D8375), 3 µM Rotenone (complex I inhibitor) (Sigma-Aldrich, R8875) and 3 µM Antimycin A (complex III inhibitor) (Sigma-Aldrich, A8674). Synovial fibroblasts were seeded at 30,000 cells/well 24 h prior to the assay. OCR and ECAR were measured five times at baseline and four times after each drug was added, with the average taken for multiple measurements.

### 4.7. Lactate Assay

The lactate concentration in cultured OA synovial fibroblasts (20,000 cells per well) was quantified using a commercially available lactate assay (MAK064-1KT Sigma-Aldrich, UK). In brief, culture supernatants were centrifuged at 12,000× *g* for 10 min in 10 kDa molecular weight cut-off spin columns (Abcam, UK) to remove proteins including lactate dehydrogenase (LDH). Absorbance was measured at 570 nm (A570) on a microplate and lactate concentration was obtained from the standard curve.

### 4.8. NMR Spectroscopy

Synovial fluids were treated with Hyaluronidase (1000 units/mL) for 15 min at 37 °C in order to decrease viscosity of samples. For the analysis of synovial fibroblasts, conditioned media were collected from cells seeded at 40,000 cells per well following 24 h either unstimulated or stimulated with TNFα at a concentration of 10 ng/mL. Samples were prepared for NMR analyses by centrifuging at 15,000× *g* for 5 min at 4 °C before being placed into 3 kDa Molecular weight cut-off (MWCO) filters (Pall Nanosep). Samples were then centrifuged at 10,000× *g* for 15 min at 4 °C. The filtrate was mixed 1:3 with NMR buffer (containing 400 mM phosphate, 1.6 mM DFTMP, 40% D2O, 0.4% azide, DSS (2 mM) at pH 7.0) and an aliquot (50 µL) added to a 1.7 mm OD NMR tube and capped. NMR spectra were acquired using a 600 MHz 4-channel Bruker AVANCE III spectrometer equipped with a Samplejet refrigerated autosampler (Bruker BioSpin, Billerica, MA, USA). For each sample, one-dimensional (1D) 1H-NMR NOSEY spectra were acquired. NMR spectra were automatically phased, and baseline corrected, and the chemical shifts were internally referenced to DSS at 0.00 ppm using Metabolab [46]. Spectral regions containing only noise and the residual water peak were excluded. Metabolites were identified and labelled using the metabolite discovery software Chenomx (Chenomx, Edmonton, AB, Canada), using the in-house library of metabolite spectra. Multivariate analysis was undertaken using SIMCA 16 (Umetrics).

## 5. Statistical Analysis

Data were analysed using GraphPad Prism 9 software (GraphPad Software, San Diego, CA, USA). For analysis of metabolite concentrations in OA patient synovial fluids, normal distribution was checked by Shapiro–Wilk normality tests. Gaussian distributions were not assumed and Mann–Whitney tests were used to examine differences between obese and normal weight values. Groups were compared using one-way analysis of variance (ANOVA) with Dunnett’s test for multiple comparisons. Data are presented as the mean ± SEM, with *p*-values < 0.05 defining statistically significant differences.

## Figures and Tables

**Table 1:** Characteristics of the patient participants for the NMR spectroscopy.

		Obese	Normal-Weight	*p*-Value
Age (years)		63 ± 4	72 ± 2.0	0.05
Female:Male (n)		5:3	5:3	
BMI (kg/m^2^)		33.6 ± 1.2	22.9 ± 0.7	<0.0001
BMI (min, max)		30.1, 38.6	20.0, 23.7	
Waist circumference (cm)		106.5 ± 5.1	86.6 ± 3.2	<0.01
Hip circumference, (cm)		116.4 ± 3.6	94.4 ± 2.1	<0.001
Waist:Hip ratio		0.91 ± 0.03	0.92 ± 0.03	0.93
KL Grade	Median (IQR)	4 (3–4)	3.5 (3–4)	
	KL3 (%)	37.5	37.5	
	KL4 (%)	62.5	62.5	
Joint Space (mm)		1.4 ± 0.7	1.8 ± 0.3	0.60

## Data Availability

Data reported in the study are available upon request to the corresponding author.

## Supplementary Materials

The following are available online at https://www.mdpi.com/article/10.3390/ijms23063266/s1.
