# Supplementary material for: Differential Metabotypes in Synovial Fibroblasts and Synovial Fluid in Hip Osteoarthritis Patients Support Inflammatory Responses
Source: Int J Mol Sci. 2022 Mar 17;23(6):3266. doi: 10.3390/ijms23063266 (PMC8950319; doi:10.3390/ijms23063266)
Supplement: Supplementary file 1 [file ijms-23-03266-s001.zip › Supplementary Table S1 NMR metabolites.pdf]

Supplementary Table S1. Metabolites detected in OA synovial fluid by NMR spectroscopy

Metabolite	NW (uM)	OB (uM)	Fold-Change	P-Value
Succinate	4.6	17.4	3.8	0.013
Lactate	899	2819	3.1	0.016
N-Nitrosodimethylamine	39.8	276	6.9	0.016
Pyruvate	28.5	93.1	3.3	0.018
Glycine	73.7	205	2.8	0.019
1,3-Dimethylurate	2.8	128	46.4	0.024
Tyrosine	19.2	59.2	3.1	0.025
Glucose	1440	4253	3.0	0.043
Phenylalanine	15.9	42.0	2.6	0.050
Valine	69.5	227	3.3	0.064
Urea	1392	3584	2.6	0.067
Acetoacetate	n.d	24.8	>100	0.067
Guanidoacetate	33.1	89.6	2.7	0.069
Methanol	34.2	n.d	>100	0.085
sn-Glycero-3-phosphocholine	7.9	172	21.7	0.093
Creatine phosphate	50.7	249.4	4.9	0.093
1,3-Dihydroxyacetone	n.d	10.0	>100	0.104
Glycylproline	n.d	176	>100	0.104
2-Hydroxyisocaproate	n.d	10.8	>100	0.104
N,N-Dimethylglycine	0.3	139	554.2	0.105
Pantothenate	3.8	436	116.2	0.107
Alloisoleucine	n.d	180	>100	0.108
Glycolate	52.3	208	4.0	0.110
Threonate	n.d	17.0	>100	0.110
Caprate	n.d	38.2	>100	0.111
Lysine	n.d	117	>100	0.116
Cadaverine	n.d	57.4	>100	0.122
2-Hydroxyvalerate	n.d	18.8	>100	0.123
Malonate	566	n.d	>100	0.125
Formate	71.8	184	2.6	0.126
5-Aminolevulinate	3.4	159	47.1	0.138
O-Phosphocholine	n.d	45.4	>100	0.153
Glutamate	47.5	563	11.8	0.154
Galactarate	22.5	192	8.5	0.162
Succinylacetone	2.8	227	82.5	0.174
Ethylene glycol	1671	n.d	>100	0.189
Alanine	65.3	192	2.9	0.194
N-Methylhydantoin	0.5	2.8	6.2	0.201
Oxypurinol	n.d	138	>100	0.206
Galactonate	30.4	n.d	>100	0.207
Hypoxanthine	1.5	7.9	5.5	0.221
3-Hydroxyisovalerate	11.7	26.8	2.3	0.230
Hydroxyacetone	7.3	n.d	>100	0.235
Riboflavin	n.d	10.0	>100	0.251
Trimethylamine N-oxide	24.2	n.d	>100	0.283
1,7-Dimethylxanthine	0.5	83.7	173.2	0.294

Metabolite	NW (uM)	OB (uM)	Fold-Change	P-Value
N-Acetylglucosamine	1.5	463	312.0	0.295
2-Oxoisocaproate	n.d	4.9	>100	0.297
Caprylate	n.d	5.5	>100	0.297
Butanone	n.d	81.9	>100	0.297
Thymidine	n.d	0.8	>100	0.297
3,5-Dibromotyrosine	n.d	1.2	>100	0.297
Methylamine	n.d	1.7	>100	0.297
UDP-N-Acetylglucosamine	n.d	2.6	>100	0.297
Nα-Acetyllysine	n.d	2.6	>100	0.297
N-Acetylaspartate	n.d	2.8	>100	0.297
Propylene glycol	n.d	3.3	>100	0.297
N-Acetylcysteine	n.d	3.5	>100	0.297
N-Acetylglutamate	n.d	4.7	>100	0.297
Choline	n.d	8.9	>100	0.297
ADP	n.d	9.2	>100	0.297
Isoleucine	n.d	10.5	>100	0.297
Methionine	n.d	15.0	>100	0.297
Histidine	n.d	15.3	>100	0.297
Aspartate	n.d	16.1	>100	0.297
Galactose	n.d	16.5	>100	0.297
Valerate	n.d	17.0	>100	0.297
Isobutyrate	n.d	21.3	>100	0.297
N-Acetyltyrosine	n.d	24.6	>100	0.297
Lactulose	n.d	40.5	>100	0.297
Ascorbate	n.d	45.2	>100	0.297
myo-Inositol	n.d	107	>100	0.297
Isocitrate	n.d	165	>100	0.297
Glucitol	n.d	170	>100	0.297
N-Acetylglycine	n.d	182	>100	0.297
Glycerate	n.d	465	>100	0.297
Lactose	n.d	662	>100	0.297
Arabinose	n.d	709	>100	0.297
Acetone	21.0	3.7	-5.6	0.305
Threonine	12.8	720	56.4	0.305
3-Hydroxybutyrate	126.8	262	2.1	0.316
3-Methylxanthine	8.4	25.0	3.0	0.332
Anserine	41.1	116	2.8	0.345
Glutamine	294.6	481	1.6	0.347
4-Hydroxy-3-methoxymandelate	6.4	0.9	-6.8	0.372
5-Hydroxyindole-3-acetate	0.6	n.d	>100	0.389
2-Hydroxyisovalerate	0.7	n.d	>100	0.389
N-Acetylserotonin	0.7	n.d	>100	0.389
Dimethylamine	1.0	n.d	>100	0.389
N-Acetylornithine	1.3	n.d	>100	0.389
Theophylline	2.4	n.d	>100	0.389
Carnosine	2.5	n.d	>100	0.389
Isopropanol	3.0	n.d	>100	0.389
N-Acetylglutamine	7.1	n.d	>100	0.389
Sebacate	8.1	n.d	>100	0.389

Metabolite	NW (uM)	OB (uM)	Fold-Change	P-Value
Ethanol	11.3	n.d	>100	0.389
Homovanillate	12.7	n.d	>100	0.389
Dimethyl sulfone	19.8	n.d	>100	0.389
Leucine	23.5	n.d	>100	0.389
Carnitine	25.4	n.d	>100	0.389
Isoeugenol	34.8	n.d	>100	0.389
cis-Aconitate	67.1	n.d	>100	0.389
2-Phosphoglycerate	302	n.d	>100	0.389
Xylose	672	n.d	>100	0.389
2-Hydroxyisobutyrate	108.0	2.0	-55.1	0.395
Acetate	38.8	66.3	1.7	0.427
Pyridoxine	1.9	7.9	4.2	0.438
Galactitol	8.9	37.2	4.2	0.439
N-Isovaleroylglycine	1.7	6.3	3.8	0.459
4-Pyridoxate	8.0	19.2	2.4	0.467
Xanthine	12.1	2.1	-5.7	0.479
Acetamide	0.9	2.9	3.2	0.499
3-Methylhistidine	9.6	19.7	2.0	0.508
Creatine	13.9	32.2	2.3	0.566
N,N-Dimethylformamide	10.6	4.0	-2.6	0.605
1-Methylhistidine	76.9	115	1.5	0.658
Syringate	0.3	0.5	1.8	0.684
Histamine	14.4	8.0	-1.8	0.706
Thymine	1.8	3.0	1.6	0.719
O-Acetylcarnitine	5.0	7.2	1.4	0.733
Trimethylamine	4.2	2.6	-1.6	0.738
3-Methyl-2-oxovalerate	24.5	14.4	-1.7	0.745
Imidazole	8.9	6.4	-1.4	0.749
Creatinine	22.3	27.7	1.2	0.757
O-Acetylcholine	15.0	20.8	1.4	0.782
3,4-Dihydroxymandelate	1.7	1.2	-1.4	0.821
Methylguanidine	1.3	0.9	-1.4	0.842
Sarcosine	9.9	7.5	-1.3	0.842
Betaine	209	165	-1.3	0.844
Caffeine	13.5	14.2	1.1	0.972
Glutaric acid monomethyl ester	33.0	34.5	1.0	0.975
2-Hydroxybutyrate	18.8	19.2	1.0	0.982
