# Supplementary material for: Differential Metabotypes in Synovial Fibroblasts and Synovial Fluid in Hip Osteoarthritis Patients Support Inflammatory Responses
Source: Int J Mol Sci. 2022 Mar 17;23(6):3266. doi: 10.3390/ijms23063266 (PMC8950319; doi:10.3390/ijms23063266)
Supplement: Supplementary file 1 [file ijms-23-03266-s001.zip › Supplementary Table S2 regression analysis.pdf]

Supplementary Table S2. Regression analysis of synovial fluid metabolite concentrations with parameters of OA patient body composition.

	BMI			WC (cm)			HC (cm)			WHR		
	R^2	Slope	p-value	R^2	Slope	p-value	R^2	Slope	p-value	R^2	Slope	p-value
1,3 Dimethylurate	0.28	9.0 ± 4.8	0.09	0.13	2.0 ± 1.8	0.30	0.13	2.9 ± 2.7	0.31	0.10	390 ± 415	0.38
Glucose	0.36	249 ± 110	0.052	0.22	60 ± 40	0.17	0.13	67 ± 62	0.31	0.30	15700 ± 8400	0.1
Glycine	0.58	13.2 ± 3.7	*0.006***	0.60	4.0 ± 1.1	*0.009***	0.39	4.7 ± 2.1	0.05	0.66	935 ± 236	*0.004***
Lactate	0.41	145 ± 61	*0.046**	0.56	50 ± 17	*0.02**	0.26	51 ± 33	0.16	0.90	14500 ± 1900	*0.0001****
N-Nitrosodimethylamine	0.36	18.4 ± 8.2	0.051	0.51	7.0 ± 2.4	*0.02**	0.34	8.4 ± 4.1	0.08	0.51	1560 ± 542	*0.02**
Pyruvate	0.32	4.8 ± 2.3	0.07	0.25	1.3 ± 0.8	0.15	0.13	1.4 ± 1.3	0.31	0.38	355 ± 161	0.059
Succinate	0.50	1.1 ± 0.4	*0.015**	0.57	0.38 ± 0.11	*0.01**	0.42	0.47 ± 0.2	*0.04**	0.52	80 ± 28	*0.019**
Tyrosine	0.39	3.4 ± 1.4	*0.046**	0.37	1.0 ± 0.47	0.06	0.20	1.1 ± 0.8	0.20	0.52	267 ± 92	*0.019**
Glutamine	0.11	-12.3 ± 14.4	0.43	0.03	-2.1 ± 4.7	0.17	0.04	-3.5 ± 6.6	0.61	0.02	-379 ± 1161	0.75
Glutamate	0.04	22.0 ± 43.7	0.63	0.29	18.4 ± 11.7	0.17	0.13	17.4 ± 18.3	0.38	0.45	5586 ± 2542	0.07
Glutamine:Glutamate	0.36	-0.20 ± 0.11	0.11	0.55	-0.08 ± 0.03	*0.036**	0.49	-0.10 ± 0.04	0.051	0.38	-15.4 ± 8.0	0.11

BMI=Body mass index, WC=waist circumference, HC=hip circumference, WHR=waist:hip ratio.
