# Supplementary material for: Differential Metabotypes in Synovial Fibroblasts and Synovial Fluid in Hip Osteoarthritis Patients Support Inflammatory Responses
Source: Int J Mol Sci. 2022 Mar 17;23(6):3266. doi: 10.3390/ijms23063266 (PMC8950319; doi:10.3390/ijms23063266)
Supplement: Supplementary file 1 [file ijms-23-03266-s001.zip › Supplementary Table S3 qPCR primers.pdf]

Supplementary Table S3: Primer sequences used for RT-PCR

Gene Symbol	Forward Sequence (5'-3')	Reverse Sequence (5'-3')	Melting Temperature (Tm)	G-C percentage (GC%)
**18s**	GTAACCCGTTGAACCCCCATT	CCATCCAATCGGTAGTAGCG	Fwd: 60.55<br>Rev: 57.93	Fwd: 52.38<br>Rev: 55.00
**IL6**	GCGCAGCTTTAAGGAGTTCCT	CCATGCTACATTTGCCGAAGA	Fwd: 60.95<br>Rev: 58.98	Fwd: 52.38<br>Rev: 47.62
**GLS1**	GCTGTGCTCCATTGAAGTGA	GCAAACTGCCCTGAGAAGTC	Fwd: 58.47<br>Rev: 59.12	Fwd: 50.00<br>Rev: 55.00
**Actin**	CCAACCGCGAGAAGATGA	CCAGAGGCGTACAGGGATAG	Fwd: 57.09<br>Rev: 59.04	Fwd: 55.56<br>Rev: 60.00
