# Supplementary figures and images for: Differential Metabotypes in Synovial Fibroblasts and Synovial Fluid in Hip Osteoarthritis Patients Support Inflammatory Responses
Source: Int J Mol Sci. 2022 Mar 17;23(6):3266. doi: 10.3390/ijms23063266 (PMC8950319; doi:10.3390/ijms23063266)

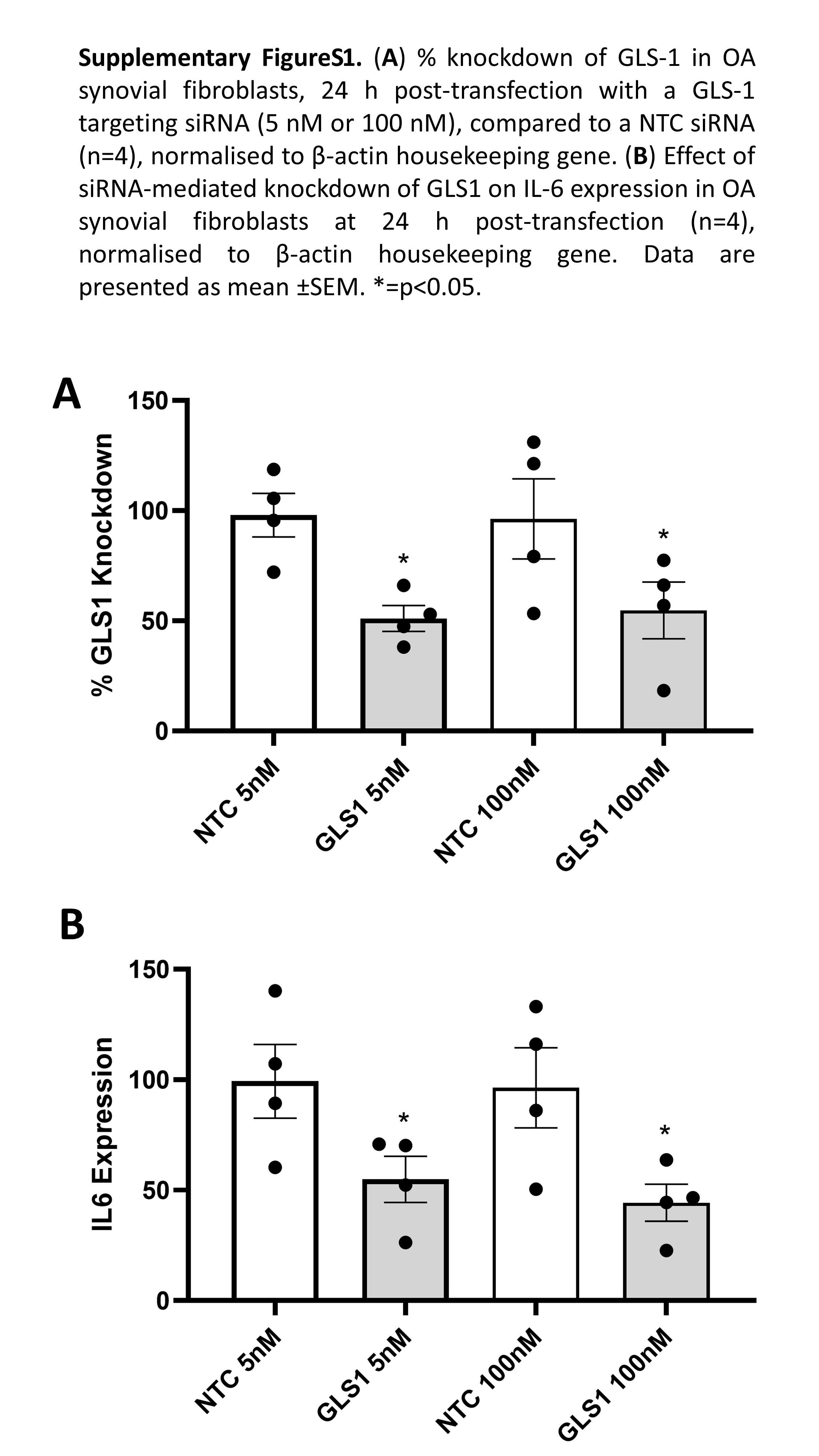

Supplement: Supplementary file 1 [file ijms-23-03266-s001.zip › Supplementary Figure S1 GLS1 and IL6 actin normalised.tif]

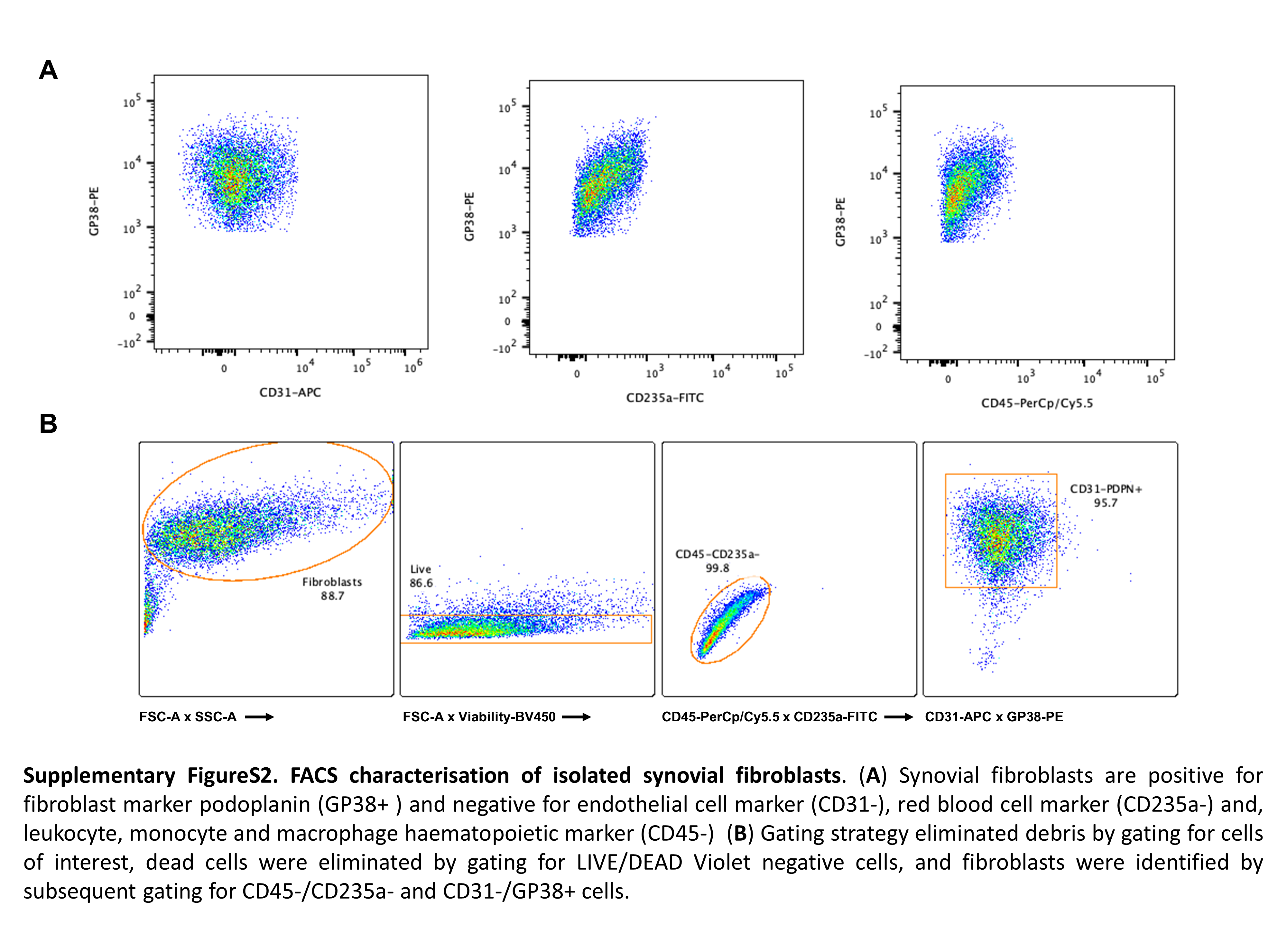

Supplement: Supplementary file 1 [file ijms-23-03266-s001.zip › Supplementary Figure S2 Fibroblast FACS.tif]
